# An Unusual Presentation of Clostridioides Difficile Colitis in a Patient on Opioids

**DOI:** 10.7759/cureus.25462

**Published:** 2022-05-29

**Authors:** Oluwafemi A Ajibola, Taiwo O Aremu, Sikder Hassan, Nili Gujadhur, Valerie Cluzet

**Affiliations:** 1 Department of Medicine, Nuvance Health, Vassar Brothers Medical Center, Poughkeepsie, USA; 2 Department of Pharmaceutical Care & Health Systems, College of Pharmacy, University of Minnesota, Minneapolis, USA; 3 Division of Environmental Health Sciences, School of Public Health, University of Minnesota, Minneapolis, USA

**Keywords:** clostridioides difficile colitis, hematochezia, constipation, diarrhea, antibiotics, opioids, colitis, clostridium difficile

## Abstract

*Clostridioides difficile* colitis is an inflammation of the colon due to toxins produced by a gram-positive bacterium called *Clostridioides difficile* (also known as *Clostridium difficile*). *Clostridioides difficile* colitis is associated with an increased risk of morbidity and mortality in elderly patients. The infection develops because of the disruption of the microbiome that usually suppresses the overgrowth of *Clostridioides difficile*. Testing for *Clostridium difficile* infection is routinely recommended in patients with at least three loose bowel movements in a day. We present an unusual case of a 74-year-old woman on chronic opioids who presented with a three-day history of lower abdominal pain, constipation, hematochezia, with no diarrhea. Radiologic imaging showed evidence of colitis, and the patient was found to have *Clostridium difficile* colitis.

## Introduction

*Clostridioides difficile* colitis is caused by toxins produced by *Clostridioides difficile* (also known as *Clostridium difficile* or *C. difficile*), a gram-positive, spore-forming, obligate anaerobic rod bacterium [1]. According to the Centers for Disease Control and Prevention (CDC), *C. difficile* causes almost 500,000 infections in the United States each year [2]. The infection is typically related to antibiotic use, usually misuse and overuse, which often characterize antimicrobial resistance [3]. The current recommendations by the American College of Gastroenterology, the Infectious Diseases Society of America, and our local health institution support testing for *C. difficile* only in patients having diarrhea (defined as three or more loose stools within 24 hours) and after holding off laxative medications [4,5]. We present an unusual case of *C. difficile* colitis in a patient on opioids with no diarrhea.

## Case presentation

A 74-year-old woman presented to the emergency department with a three-day history of lower abdominal pain, constipation, and blood in the stool (hematochezia) four days after a recent hospitalization for community-acquired pneumonia. She has a past medical history of anxiety, depression, chronic obstructive pulmonary disease, hypertension, hyperlipidemia, coronary artery disease, gastroesophageal reflux disease, mild-moderate mitral regurgitation, hypothyroidism, systemic lupus erythematosus, substance use disorder on methadone maintenance, and constipation. Before her recent discharge from the hospital, she was treated with ceftriaxone and azithromycin.

On admission, she had a low-grade temperature of 99.4°F, blood pressure of 137/73 mmHg, respiratory rate of 20/minute, heart rate of 70 beats per minute, and oxygen saturation of 100% on room air. Physical examination was remarkable for dry mucous membranes and abdominal tenderness in the left upper and lower quadrants. Rectal examination showed a normal tone with frank blood. Laboratory studies revealed leukocytosis with absolute neutrophilia, normal hemoglobin level, thrombocytosis, elevated blood urea nitrogen and creatinine, hyponatremia, hypokalemia, and normal serum lactic acid levels (Table 1).

**Table 1:** Laboratory values

Laboratory parameter	Patient’s value	Reference range
White blood cell count	17.9 k/µL	3.5–10 k/µL
Neutrophils	83%	40–75%
Hemoglobin	11.5 g/dL	12–16 g/dL
Platelet count	197 k/mm^3^	150–400 k/mm^3^
Blood urea nitrogen	25.8 mg/dL	6–20 mg/dL
Creatinine	1.86 mg/dL	0.4–1mg/dL
Sodium	131 mmol/L	136–145 mmol/L
Potassium	5.5 mmol/L	3.5–5.1 mmol/L
Lactic acid	1 mmol/L	≤2.0 mmol/L

**Figure 1:**
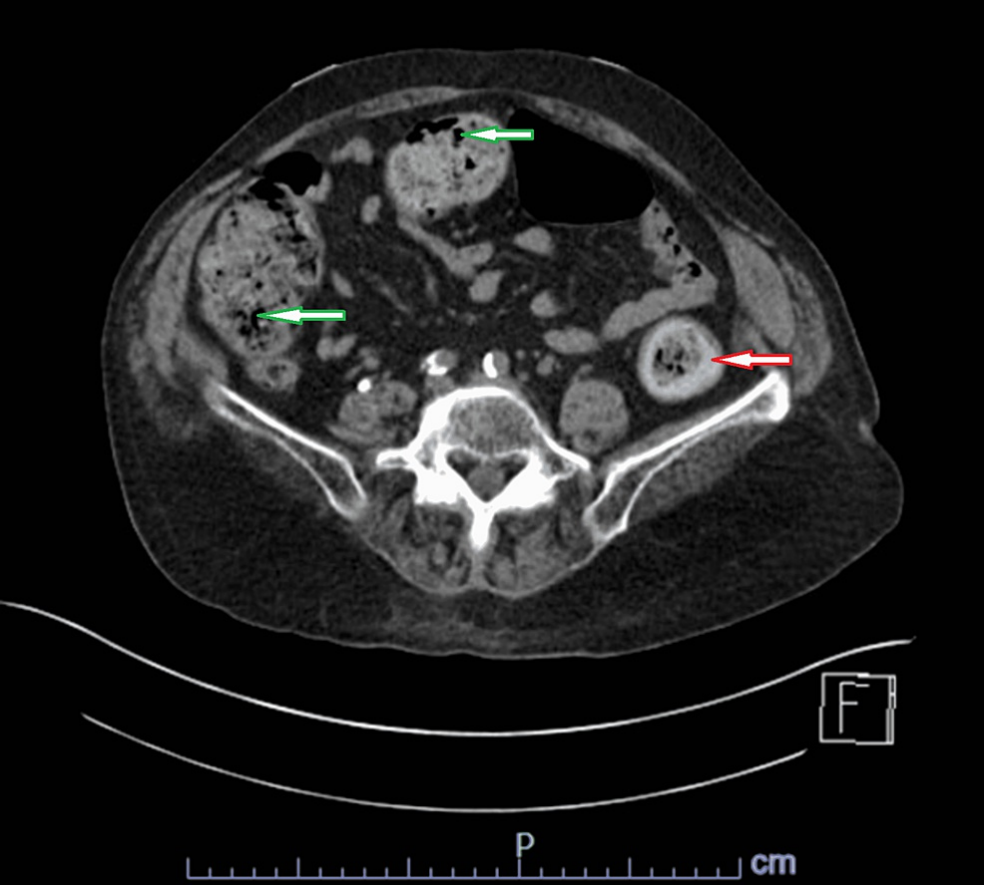
CT of the abdomen and pelvis without contrast showing a moderate amount of stool throughout the colon, and mural thickening in the rectum and proximal sigmoid colon. The green arrows indicate areas of moderate amount of stool in the colon. The red arrow indicates an area of mural thickening.

A non-contrast computed tomography (CT) scan of the abdomen and pelvis showed a moderate amount of stool throughout the colon, suggesting constipation and mural thickening in the rectum and proximal sigmoid colon concerning for colitis (Figure 1).

**Figure 2:**
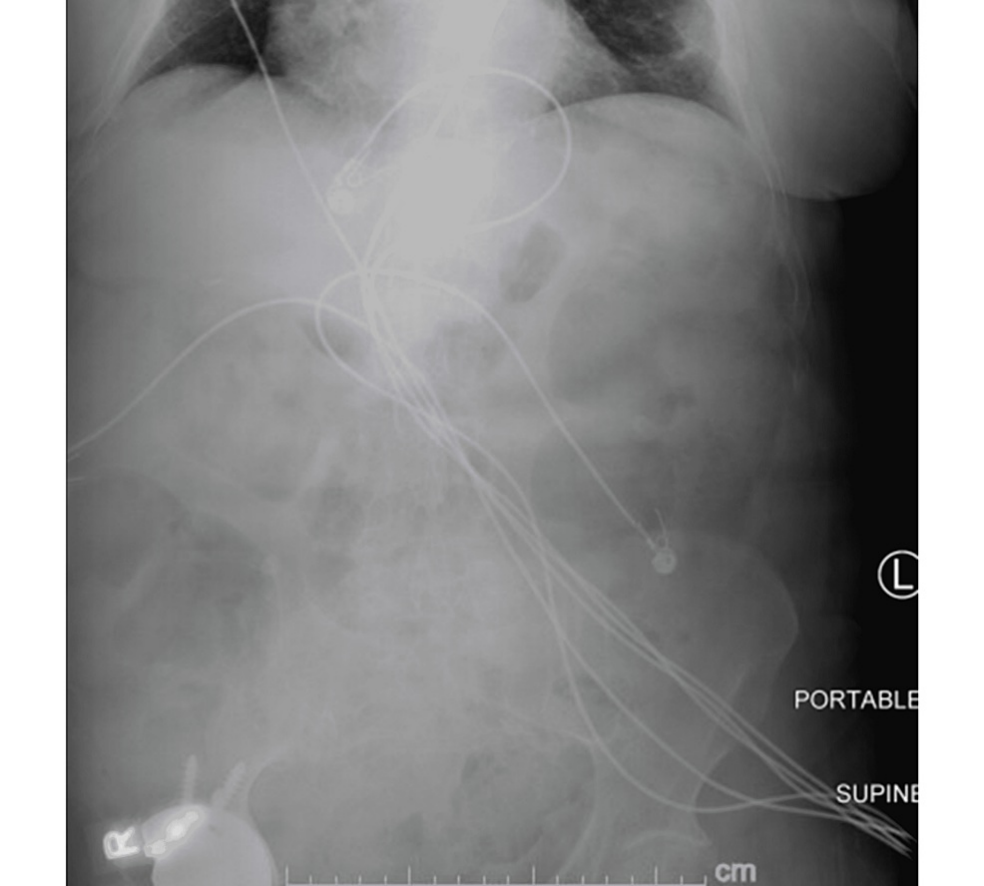
Abdominal X-ray showing no evidence of free intraperitoneal air, dilated colon, or obstruction.

While in the emergency department, the patient had a single loose stool with brown and bloody components. She was started on intravenous fluids and broad-spectrum antibiotics, namely ceftriaxone and metronidazole. Due to recent antibiotic exposure and evidence of colitis on the CT scan, the patient was tested for *C. difficile*. Both *C. difficile* DNA PCR and toxin were positive. The patient was then placed on contact precautions, parenteral antibiotics were discontinued, and she was started on oral vancomycin. On day 5 of hospitalization, an abdominal X-ray was performed to evaluate abdominal distension, which showed no evidence of free intraperitoneal air, dilated colon, or obstruction (Figure 2).

She was treated for methadone-induced constipation with methylnaltrexone bromide. Subsequently, her abdominal pain, leukocytosis, and renal failure improved, and hematochezia resolved. The patient was discharged home on day 8 in stable condition.

## Discussion

*Clostridioides difficile* has a wide range of clinical manifestations, from asymptomatic colonization to fulminant disease with toxic megacolon. *Clostridioides difficile* infection is associated with an increased risk of morbidity and mortality in the elderly (aged 65 years and older) [2]. Besides, around 9% of the elderly who have been diagnosed with healthcare-associated *C. difficile *infection die within one month [2]. In addition to pneumonia and surgical site infections, *C. difficile* infection is a leading cause of nosocomial infection in the United States [6-8]. In 2017, hospitalized patients accounted for around 223,900 cases of nosocomial *C. difficile* and around 12,800 deaths in the United States [9].

The normal microbiota of healthy intestines often suppresses the growth of *C. difficile*, and disruption of the microbiome can lead to the overgrowth of the bacteria [10,11]. The *C. difficile* bacterium produces toxins A and B that disrupt the cytoskeleton of the colon, hence leading to pseudomembranous colitis [11]. Risk factors for *C. difficile* infection include exposure to antibiotics, age 65 years and older, recent hospitalization or nursing home stay, immunosuppression (including HIV/AIDS, cancer, and organ transplantation), immunosuppressive therapies, hypoalbuminemia, and previous *C. difficile* infection [2,11]. Symptoms include diarrhea (defined as at least three loose bowel movements in a day), fever, abdominal pain, nausea, and loss of appetite.

The American College of Gastroenterology, the Infectious Diseases Society of America, and our institutional guidelines recommend only testing for *C. difficile* in patients with diarrhea [4,5]. In our case, the patient did not present with diarrhea and did not meet the criteria for testing based on the current guidelines. It is important to note that the patient was on methadone maintenance, a long-acting opioid with constipation as one of its major side effects. In addition, elderly individuals are also at an increased risk of constipation due to decreased gastrointestinal motility. The side effect of this medication and the patient's age likely masked the usual symptoms of *C. difficile* colitis, specifically diarrhea. Although the patient did not meet the criteria for testing based on stool frequency, she warranted testing given other consistent symptoms, recent antibiotic exposure, markedly elevated white blood cell count, and evidence of colitis on the abdominal CT scan.

## Conclusions

*Clostridioides difficile* infection is associated with significant morbidity and mortality in individuals aged 65 years and older. The early diagnosis of *C. difficile* colitis can decrease the risk of transmission, length of hospital stays, and mortality. Although the current recommendations do not support testing patients who present without diarrhea, suspicion for *C. difficile* infection should still be maintained in patients on anti-motility agents who have other clinical signs and symptoms consistent with *C. difficile* colitis.
